# Into Thick(er) Air? Oxygen Availability at Humans' Physiological Frontier on Mount Everest

**DOI:** 10.1016/j.isci.2020.101718

**Published:** 2020-11-20

**Authors:** Tom Matthews, L. Baker Perry, Timothy P. Lane, Aurora C. Elmore, Arbindra Khadka, Deepak Aryal, Dibas Shrestha, Subash Tuladhar, Saraju K. Baidya, Ananta Gajurel, Mariusz Potocki, Paul A. Mayewski

**Affiliations:** 1 Department of Geography & Environment, Loughborough University, Loughborough, UK; 2 Department of Geography & Planning, Appalachian State University, Boone, NC, USA; 3 School of Biological and Environmental Sciences, Liverpool John Moores University, Liverpool, UK; 4 National Geographic Society, Washington, D.C, USA; 5 Central Department of Hydrology & Meteorology, Tribhuvan University, Kathmandu, Nepal; 6 International Centre for Integrated Mountain Development, Lalitpur, Nepal; 7 Department of Hydrology and Meteorology, Kathmandu, Nepal; 8 Department of Geology, Tribhuvan University, Kathmandu, Nepal; 9 Climate Change Institute, University of Maine, Orono, ME, USA; 10 School of Earth and Climate Sciences, University of Maine, Orono, ME, USA

**Keywords:** Physiological State, Physical Activity, Climatology, Atmospheric Observation, Glacial Landscapes

## Abstract

Global audiences are captivated by climbers pushing themselves to the limits in the hypoxic environment of Mount Everest. However, air pressure sets oxygen abundance, meaning it varies with the weather and climate warming. This presents safety issues for mountaineers but also an opportunity for public engagement around climate change. Here we blend new observations from Everest with ERA5 reanalysis (1979-2019) and climate model results to address both perspectives. We find that plausible warming could generate subtle but physiologically relevant changes in summit oxygen availability, including an almost 5% increase in annual minimum VO_2_ max for 2°C warming since pre-industrial. In the current climate we find evidence of swings in pressure sufficient to change Everest's apparent elevation by almost 750 m. Winter pressures can also plunge lower than previously reported, highlighting the importance of air pressure forecasts for the safety of those trying to push the physiological frontier on Mt. Everest.

## Highlights

• Mt. Everest's perceived elevation changes by almost 700 m depending on the weather
• Air pressure on Mt. Everest summit plunges close to physiological limit in winter
• Climate warming is increasing air pressure/oxygen availability on Mt. Everest

## Graphical Abstract

**Figure:**
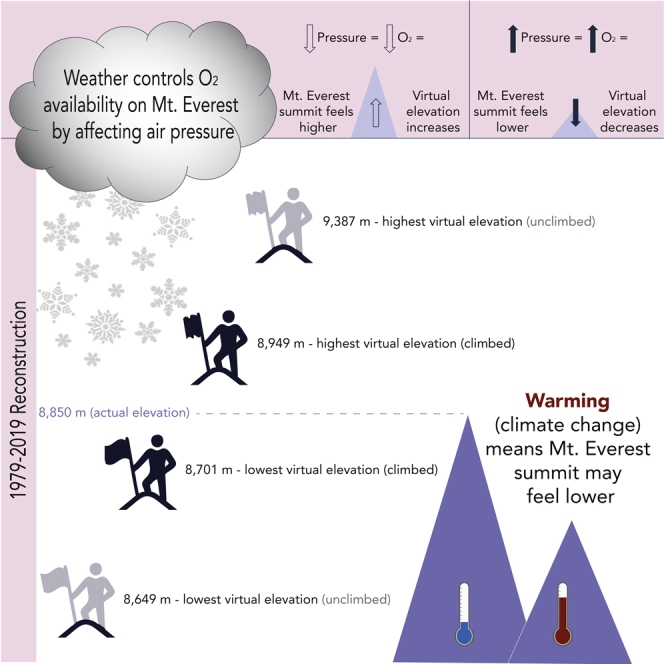

## Introduction

As the highest mountain on Earth, Mt. Everest is one of the planet's most extreme environments. First climbed almost half a century after the North and South Poles had been reached, the 8,850 m peak (known in Nepal and China as Sagarmatha and Qomolangma, respectively) can experience temperatures as low as −50°C and winds as high as 80 m s^−1^, placing mountaineers at risk of cold injury in as little as one minute (Huey and Eguskitza, 2001; Matthews et al., 2020; Moore and Semple, 2011). Yet it is the dangers from reduced oxygen availability that provides perhaps the greatest challenge to climbers (West, 2019). In that sense the summit of Mt. Everest is a remarkable frontier, perhaps closer than anywhere else on the Earth's surface to the limits where human physiology can reach.

Oxygen content is so low near the summit of Mt. Everest because its abundance is directly proportional to atmospheric air pressure within the troposphere, and this falls exponentially with increasing elevation. The lower oxygen availability challenges many organ functions (Richalet, 2010; West, 1990), but it is the reduction in potential work rate that so greatly tests mountaineers (West, 1993). Empirical studies have shown that, even in acclimatized individuals, aerobic capacity (expressed by maximal oxygen uptake: VO_2_ max) declines exponentially with falling air pressure (Pugh et al., 1964; Sutton et al., 1988; West et al., 1983a). Mt. Everest's summit is so close to the limits of human physiology that the earliest of these studies concluded it could not be reached without climbers breathing bottled oxygen (Pugh et al., 1964; West, 2019). It was only when Reinhold Messner and Peter Habeler summited on May 8, 1978 without supplemental oxygen (an “oxygenless ascent” hereafter) that the highest point on Earth was confirmed as within reach of human physiology. Although theoretical analyses struggled to explain this result, later work concluded that, if a climber's VO_2_ max at sea level was sufficiently high, the decline with altitude may not be sufficient to preclude an oxygenless ascent (West et al., 1983a; West and Wagner, 1980).

Critically, it has been recognized that the *relatively* high air pressure on Mt. Everest also plays a key role in enabling oxygenless ascents to 8,850 m. Estimates from radiosondes indicate monthly mean summit air pressures between 324 hPa in winter and 339 hPa in the monsoon (West et al., 1983b). These values are much higher than the 313 hPa predicted by ICAO Standard Atmosphere (an industry-standard model of the pressure-height relationship), meaning that the physiological challenge of an oxygenless ascent is more manageable than would be inferred from the ICAO prediction (West and Wagner, 1980).

Air pressure is relatively high on Mt. Everest because the rate at which pressure falls with elevation is inversely proportional to the virtual temperature of the atmosphere (Stull, 2015), and Mt. Everest is, along with all other peaks over 8,000 m, located in the warmth of the subtropics. Oxygenless ascents of Earth's highest mountains would be an even greater demand for human physiology if they were located in colder climates (West, 1993, 2010; West and Wagner, 1980). There is also a temporal equivalent of this geographical good fortune that helps place Mt. Everest's summit within reach. Due to variations in the atmosphere's oxygen content through geological time, a hypothetical human would *not* have been able to ascend to 8,850 m for more than two-thirds of the last 570 million years (Huey and Ward, 2005). Similarly, given current rates of tectonic uplift, it has been estimated that Mt. Everest's summit could be beyond the limits of human physiology around 40,000 years from now (Bailey, 2001). However, in the shorter term, anthropogenic climate change is increasing the air pressure on the summit of Mt. Everest as temperatures rise (Moore and Semple, 2009) (because this causes air pressure to fall less rapidly with height). The potential aerobic difficulty of an oxygenless ascent has consequently decreased since the early 20th century and is likely to diminish further with continued warming.

The challenge of climbing to 8,850 m is therefore variable from an oxygen-availability standpoint. By coincidence of geography, atmospheric composition, and climate, it is currently within human aerobic capacity to reach this height without supplemental oxygen. However, the current proximity of Mt. Everest's summit to our physiological frontier is still somewhat unknown. Radiosonde estimates have captured the statistics of summit pressure variability (including the conditions encountered during a handful of famous ascents) (West, 1993), but a systematic, high-resolution assessment of air pressure at the summit is not yet available. Such a contribution would identify the full range of conditions that humans have encountered during past oxygenless ascents and would also clarify how close the summit may encroach upon our aerobic limits during the depths of winter when air pressure is lowest (West et al., 1983b). The latter is of critical relevance to the interest in oxygenless wintertime ascents, highlighted by multiple attempts in the 2019–2020 winter season (Benavides, 2019, 2020).

Beyond the simple upward extrapolation of the trend in summit air pressure by Moore and Semple (2009), an assessment of the potential shifts in the aerobic challenge of Mt. Everest due to climate change is also yet to be undertaken. This is of considerable symbolic and practical significance. Mt. Everest is a global icon symbolizing life at the extremes (Frohlick, 2003), and it has been argued that using tangible and specific examples can help communicate the abstract concept of climate warming (Höijer, 2010). The evolving aerobic difficulty for humans scaling Mt. Everest without supplemental oxygen is, therefore, a topic with the potential to increase public interest in climate change and perhaps improve support for policy response (Smith and Leiserowitz, 2014). The aims of our research are consequently two-fold: (1) to produce a high-resolution reconstruction of air pressure (and thus oxygen availability) on the summit of Mt. Everest, including assessing climbers' experience and exploring extreme low-pressure events; and (2) to investigate the sensitivity of summit air pressures to changes in global mean air temperature.

## Results

### Observations and Reconstructed Air Pressure

**Figure 1:**
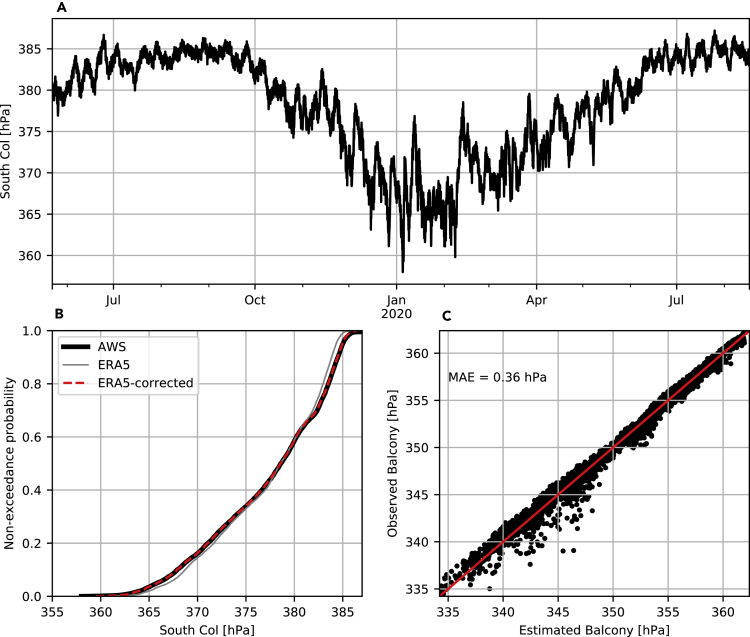
Air Pressure on Mt. Everest (A) Hourly observed air pressure at the South Col (7,945 m) from May 22, 2019 to July 1, 2020. (B) Comparison between observed air pressure and ERA5 air pressure for the South Col. (C) Estimated air pressure at the Balcony (8,430 m) compared with observed air pressure at the Balcony AWS for the time period of May 23, 2019 to January 20, 2020. MAE indicates the mean absolute error for this comparison.

Observed air pressure at the South Col automatic weather station (7,945 m) on Mt Everest (Matthews et al., 2020) shows a distinct seasonal cycle, ranging from a minimum of 358 hPa in January 2020, to a maximum of 387 hPa in June 2019 (Figure 1A). The variation in air pressure is much more substantial in winter than during the summer, with a maximum range of 20 hPa across December 2019 to February 2020, compared with a maximum range of 6 hPa during the 2019 monsoon (July–September: Matthews et al., 2020). We also note from Figure 1A that pressure is subject to relatively large changes over short time scales, exemplified by the swing of almost 20 hPa between February 8 and 13, 2020.

Overlapping data from the ERA5 reanalysis (Hersbach et al., 2020) indicate excellent agreement with South Col air pressure measurements, yielding a Pearson correlation of 0.995 and minimal bias, reduced further by application of the adjustment described in the Methods (Figure 1B). Combined with our method to estimate the vertical gradient in (log) pressure, this correction results in a mean absolute error (MAE) of 0.36 hPa when ERA5 air pressure is extrapolated from the South Col to the Balcony automatic weather station at 8,430 m (Matthews et al., 2020) (Figure 1C). By comparison, an MAE of 6.45 hPa is obtained if ERA5 data are interpolated directly to the Balcony.

**Figure 2:**
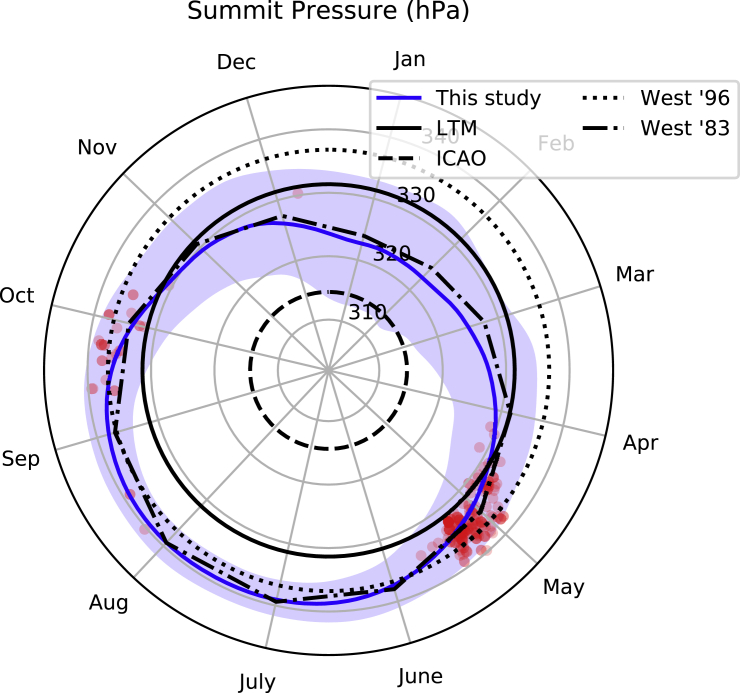
Reconstructed Air Pressure for the Summit of Mt. Everest (1979–2019) Blue shading spans the reconstructed minimum and maximum for the respective day of year, whereas the solid blue line indicates the mean value for the day. The solid black line (LTM) is the long-term mean for our reconstruction (331 hPa). The other lines appearing in the legend highlight estimates of summit pressure used in the literature (ICAO: ICAO Standard Atmosphere; West ’83: West et al. (1983b); West ’96: West (1996)). The red circles indicate summit air pressure at the time of successful ascents made without the help of supplemental oxygen (with intensity of shading proportional to the number of climbers). Note that labels are located at the middle of the respective month and that all day-or-year statistics are smoothed with a Gaussian kernel before plotting (see Methods).

The strong coherence between measured and estimated pressure at the Balcony builds confidence in our ERA5 reconstruction of summit air pressure (Figure 2), where we compute a mean value of 331 hPa over the 1979–2019 period. In agreement with earlier research (West et al., 1983b) we find substantial seasonality, as mean summit pressure ranges from 323 hPa (mid-January) to 339 hPa (mid-August). Consistent with the observational record from the South Col, the width of the blue shading in Figure 2 also highlights that summit pressure is most variable in winter.

**Figure 3:**
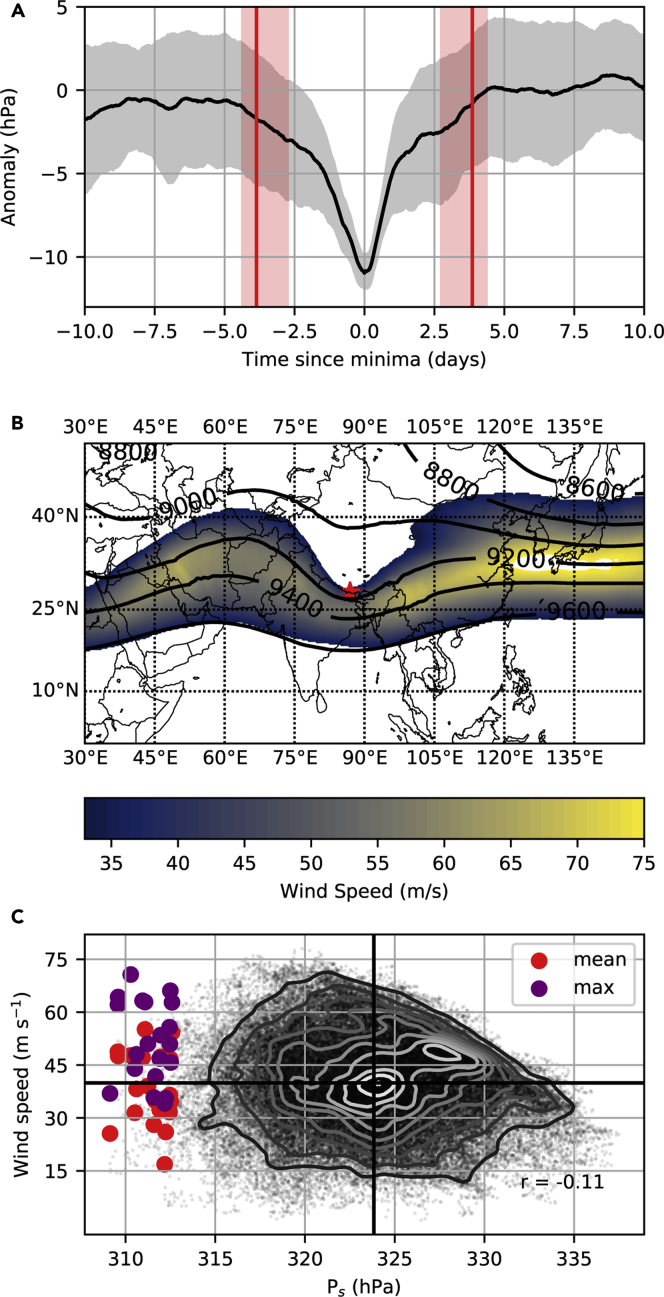
Episodes of Lowest Air Pressure on Mt. Everest (1979–2019) (A) Composite mean anomaly (black line) ± standard deviation (gray shading) for the 20 reconstructed events with lowest summit pressure. Red vertical lines mark the mean calculated time since minima for pressure to begin falling away from (negative days), or recover to (positive days), higher values. Calculations are based on wave phase speed, and the shading spans the 25^th^ to 75^th^ percentiles of these estimates (see Methods). (B) Mean geopotential height of the 300 hPa surface (lines) and mean 250 hPa wind (shading) across the 20 events, with Mt. Everest shown as a red star. Note winds <33 m/s (equivalent to a Category 1 hurricane) are not shown. (C) Scatter cloud shows the relationship between hourly winter (Dec-Feb) summit air pressures and concurrent winds interpolated to Mt. Everest's summit. Contours indicate relative density (white higher density, black lower density), whereas heavy black lines indicate the respective means, and *r* is the Pearson correlation. The larger colored circles show the mean (red) and maximum (purple) summit wind speed and pressure in a 72-h window centered on each of the 20 events.

The episodes of lowest air pressure are explored in more detail in Figure 3A, where we composite 10 days of air pressure either side of the 20 events with lowest pressure. These local minima fall to values around 10 hPa below the seasonal mean over approximately three to five days, before recovering at a similar rate. The atmospheric circulation during the low-pressure events indicates the presence of an upper-level wave, with its ridge crest over Central Asia, and its trough centered over Mt. Everest's summit (Figure 3B). Waves consistent with the composite in Figure 3B are observed in nearly all of the 20 events (Figure S1), and estimates of their phase speed (see Methods) predict a median transit time from ridge to crest of 3.9 days, in close agreement with the timescale of pressure variability evident in Figure 3A.

The strong upper atmosphere winds of the subtropical jet stream are also evident in Figure 3B, although they are focused to the south of the Himalayas. The relationship between ERA5 winds interpolated to the summit of Mt. Everest is weak during December–February (when all 20 low-pressure events occur; r = −0.11), and winds persistently below the wintertime mean are possible even during the extreme low pressure events (Figure 3C). Consistent with the understanding that air pressure falls more rapidly with elevation in colder air masses (see Introduction), we do note a strong correlation between wintertime air temperatures and summit air pressure (r = 0.81).

### Air Pressures Encountered during Oxygenless Summits

Over the period of our reconstruction (1979–2019) there have been 10,068 successful ascents of Mt. Everest, and 208 (2.1%) of those were made without the assistance of supplemental oxygen. The vast majority of these oxygenless ascents (81.7%) were achieved in the pre-monsoon month of May, with the next most popular month being October (11.1%) during the post-monsoon (Table 1). Both of these months experience summit pressures above the annual mean approximately three-quarters of the time. Accordingly, the mean summit pressure across all oxygenless summits (335 hPa) is around 4 hPa above the long-term mean (331 hPa). However, we highlight that this value is still somewhat below the 337 hPa often used by physiologists to characterize Mt. Everest's summit pressure (see Figure 2, “West ‘96”)

**Table 1:** Monthly Air Pressure Statistics and Successful Mt. Everest Ascents Made without Supplemental Oxygen (1979–2019)

Month	Summits	Summits (% of Total)	Mean Air Pressure (hPa)	Air Pressure Range (hPa)	Exceeding Annual Mean (%)^a^	Exceeding Climbing Mean (%)^b^	Mean Climbing Air Pressure^c^	Mean Climbing Air Pressure Percentile^d^
Jan	0	0	323	26	2.3	0.1	–	–
Feb	0	0	323	27	1.5	0.1	–	–
Mar	0	0	325	23	3.8	0.2	–	–
Apr	4	1.9	329	21	19.6	1.5	330	64.5
May	170	81.7	333	17	78.6	27.1	335	70.8
Jun	2	1.0	337	14	99.8	92.3	334	4.9
Jul	0	0.0	339	8	100.0	100.0	–	–
Aug	3	1.4	339	8	100.0	100.0	339	54.0
Sep	5	2.4	338	13	99.9	96.2	338	33.5
Oct	23	11.1	333	17	73.8	32.5	336	79.3
Nov	0	0.0	329	20	25.4	2.7	–	–
Dec	1	0.5	326	27	9.0	0.8	330.3	86.1

a The percentage of hours within the month when air pressure is above the long-term mean (331 hPa).

b The percentage of hours in the month when summit air pressure is above the mean across all oxygenless ascents (335 hPa).

c The mean summit air pressure during all successful oxygenless ascents made in the respective month.

d The corresponding percentile of the mean in c with respect to all summit air pressures in that month.

Successful oxygenless summits were also obtained on days with relatively high oxygen availability for the time of year (Figure 2 and Table 1), with a mean anomaly (relative to the day of year) of 1.0 hPa across all climbs and mean summit pressures during climbs in May and October equivalent to approximately the 70^th^ and 80^th^ percentiles in the respective months. The only winter climb without supplemental oxygen was also attained at a time when pressure was 330 hPa (in close agreement with the 329 hPa reported by West, 1993), which is higher than over 86% of all other December values, and close to the long-term mean (331 hPa). Although very rare, summit air pressures in all winter months (December–February) can rise above the long-term mean, even exceeding the mean pressure during oxygenless summits (0.1–0.8% of the time). The very few oxygenless summits during the normal monsoon months (June–September) took place on days with absolute values either above, or very close to, the mean pressure across all oxygenless summits.

**Figure 4:**
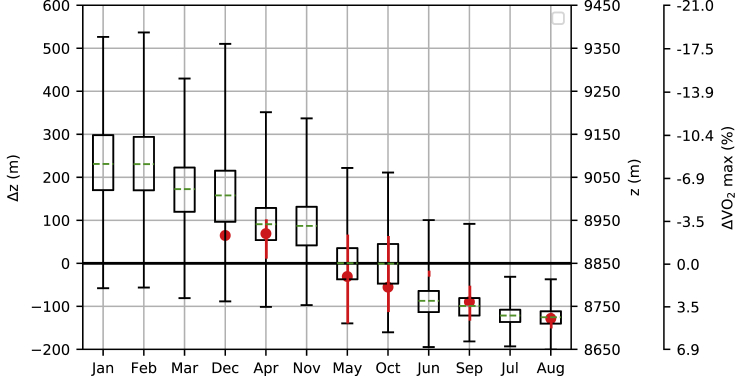
Monthly Variations in the Aerobic Impacts of Oxygen Variability at Mt. Everest's Summit For each month, Δz is the change in elevation required to reach the respective air pressure, assuming a gradient in log pressure equal to the May mean (0.00014 m^−1^) and a mean May summit pressure of 333 hPa. Bars extend to the highest and lowest Δz (corresponding to the lowest and highest summit pressures, respectively), whereas boxes span the 25^th^–75^th^ percentiles, and green lines mark the monthly mean. Note that z (m) is the total perceived summit elevation (Δz + 8,850 m), and ΔVO_2_ max converts the respective air pressures to differences in VO_2_ max, relative to the mean May conditions (333 hPa = 16.2 mL min^−1^ kg^−1^). Months are sorted in descending order of mean Δz. Red circles indicate conditions during oxygenless summits, with vertical lines extending to maximum and minimum values [For two summits only the range is plotted; and for one summit the value is denoted by the red circle]. See Supplemental Information for apparent elevation data during previous oxygen-assisted summits.

Successful ascents made without supplemental oxygen have occurred during a narrow range of the air pressures that are possible on the summit of Mt. Everest. Similar to Huey and Ward (2005) we communicate this by converting the variations in air pressure to changes in elevation for a typical May day (Figure 4). These metrics indicate how far above (or below) the summit a mountaineer would have to theoretically climb to encounter the respective air pressures. According to these definitions, the hypothetical May mountaineer would have to ascend 97 m beyond the summit to reach a level equivalent to the lowest pressure encountered during any climb without supplemental oxygen (329 hPa; April 1985); they would need to *descend* 150 m to match the highest pressure for an oxygenless summit (340 hPa, August 1980). This difference (247 m) is, however, much less than the 737 m separation calculated between the highest (343 hPa; August 2010) and lowest (309 hPa; February 1993) summit air pressures estimated throughout the reconstruction.

Another way to communicate the environmental effects on a climber at extreme elevations is through the aerobic impact (i.e., the potential VO_2_ max: see Methods) at Mt. Everest's summit (Figure 4). Across all oxygenless summits, we calculate that VO_2_ max varied between −3.4 (April 1985) and 5.1% (August 1980) of the mean May value, widening to −18.8 (February 1993) and 6.9% (August 2010) across all air pressures in the reconstruction. Relative comparisons are more striking, with estimated VO_2_ max on the summit 8.1% lower during the oxygenless summit on 29 April 1985 compared with the successful climb without supplemental oxygen in August 1980. Across the entire reconstruction, conditions during the February 1993 low-pressure episode would cut VO_2_ max by 24.0% of the value estimated for the highest-pressure event in August 2010. For context, to reduce VO_2_ max by the same fraction (according to the ICAO Standard Atmosphere) would necessitate an ascent from sea level to 3,169 m. Communicated in terms of work rate, we note that climbing speed under this February lowest pressure event could be reduced by around 41.2% relative to the highest pressure. In an illustrative example, this would result in a climber with a mass of 100 kg (including all equipment) being reduced from an elevation gain of 3.5 m min^−1^ to 2.1 m min^−1^, almost doubling the time required to climb a given distance. Compared with the lowest pressure experienced during the April 1985 oxygenless summit, the February 1993 low-pressure extreme would lower VO_2_ max by 15.9%, equivalent to the reduction expected from climbing from sea level to 2,132 m in the ICAO Standard Atmosphere. The corresponding drop in climbing speed would be 29.5% (see Methods for calculations of climbing rates).

### The Impact of Climate Warming on Summit Air Pressure

**Figure 5:**
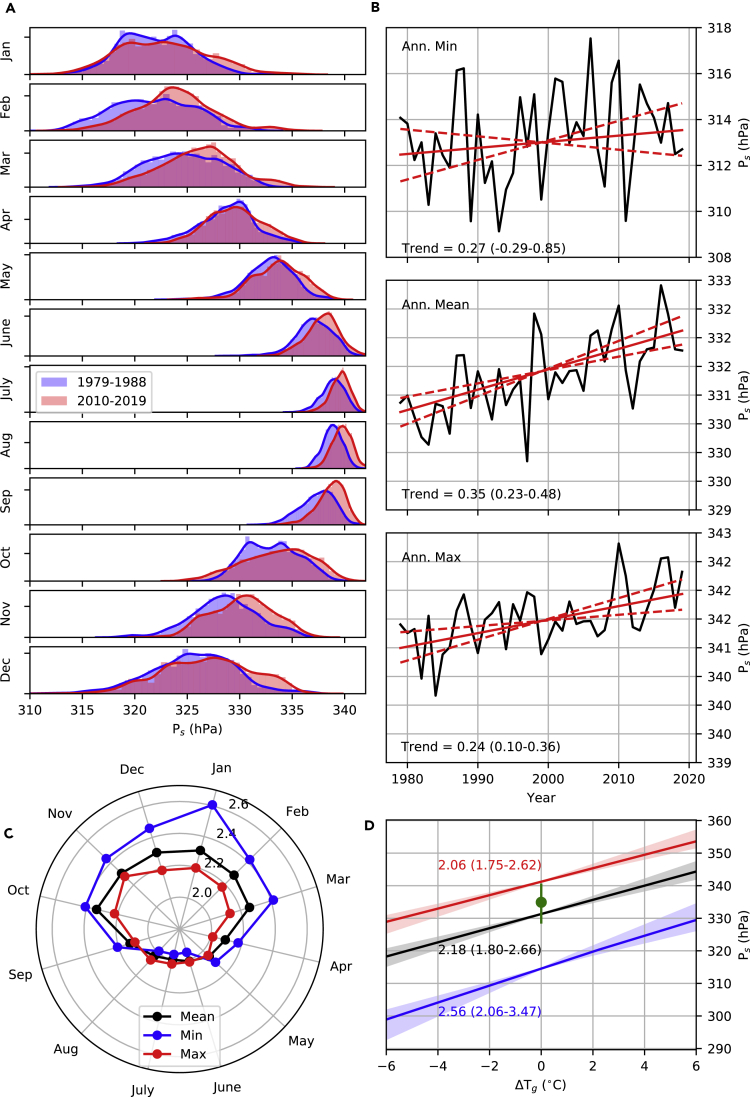
Analysis of Seasonal Air Pressure Changes at Mt. Everest's Summit (A) Observed distribution of hourly air pressure in the first and last decades of the reconstruction. (B) Observed trends in annual statistics. The solid red line indicates the median Theil-Sen slope estimate, and the dotted red lines show the 5^th^–95^th^ percentiles. Decadal trends (and uncertainty range) are annotated at the bottom of each panel. (C) CMIP5 ensemble median sensitivities of monthly statistics to global mean temperature change (hPa °C^−1^). (D) CMIP5 projections for annual mean, maximum, and minimum summit pressures for $\Delta T_{g}$of warming above 1981–2010 global mean temperature Solid lines indicate the CMIP5 ensemble median, whereas shading spans the 5^th^–95^th^ percentiles. Note that the colors share the same meaning as panel (C), and annotations summarize the gradients (hPa °C^−1^) of the lines plotted (5^th^–95^th^ percentiles). The green circle marks the mean summit pressure across all successful oxygenless ascents, with green lines extending to the minimum and maximum pressures.

A shift toward higher summit pressure in the most recent decade is visually evident across the distribution in most months (Figure 5A). This change is, however, clearest during the monsoon months of June–September when variability is reduced. During most of the winter and the popular spring climbing month of May, there is some evidence for an increase in the higher quantiles, but not the lower quantiles, indicating a broadening of the distribution. This picture is supported by trends in the annual statistics of summit air pressure (Figure 5B). Rates of change in annual mean summit pressure [0.35 (95% confidence interval: 0.23–0.48) hPa decade^−1^] and the annual maximum summit pressure [0.24 (0.10–0.36) hPa decade^−1^] are significant, according to the non-parametric Theil-Sen trend estimation, whereas the trend in annual minimum summit pressure [0.27 (−0.29 to 0.85) hPa decade^−1^] is not. The HadCRUT4 dataset (Morice et al., 2012) indicates that global mean annual air temperature increased at a rate of 0.17°C decade^−1^ over the same 1979–2019 interval, meaning that the air pressure trends equate to temperature sensitivities of 2.10 (1.38–2.85), 1.39 (0.58–2.14), and 1.58 (−1.73 to 5.03) hPa °C^−1^ for the annual mean, maximum, and minimum pressures, respectively.

The ensemble of CMIP5 simulations (Taylor et al., 2011; Table S1) offer an alternative estimate of the sensitivity of summit pressure, isolating the impact of climate warming by filtering out interannual variability through the application of running 30-year means. The results show that the largest increases should be in wintertime pressure as global mean annual air temperature rises, with minimum values most sensitive to warming (Figure 5C). This translates to greatest increases in annual minimum summit pressure, rising by 2.56 (2.06–3.47) hPa °C^−1^ compared with sensitivities of 2.06 (1.75–2.62) and 2.18 (1.80–2.66) hPa °C^−1^ for annual mean and maximum summit pressures, respectively (Figure 5D).

## Discussion

Due to a coincidence of geography, atmospheric composition, and climate, it is currently within the range of human physiology to reach the 8,850 m peak of Mt. Everest without supplemental oxygen. This is, however, more the exception than the rule for the last 570 million years (Huey and Ward, 2005), and the transience of Mt. Everest's summit being within our grasp provides a powerful way to communicate environmental change at the extremes of our planet's climate. It is also significant from a practical perspective to those capturing the world's attention with their attempts to summit Mt. Everest without supplemental oxygen (Benavides, 2019, 2020). Here we have provided the highest resolution estimate to date of how close Mt. Everest summit oxygen availability encroaches upon human aerobic limits and the most detailed assessment yet of the impact of climate change in pushing back this frontier.

Our analysis of sensitivities from climate model experiments indicated that plausible scenarios of global warming could result in air pressure changes on Mt. Everest that may be of physiological relevance. A temperature increase consistent with the 2°C limit agreed in the Paris Climate Agreement (UNFCCC, 2015), for example, could increase air pressure by 4–5 hPa relative to pre-industrial conditions (Figure 5C), translating into median changes in VO_2_ max of up to 4.9% for annual minimum pressure and 3.3% for annual means. As an example, this would translate to the minimum pressure encountered in our reconstruction (309 hPa) being expected to rise to 314 hPa, reducing the height that a hypothetical mountaineer would need to climb to reach this pressure by around 118 m (from 9,387 m to 9,269 m). Much larger and unlikely (but not implausible; Sherwood and Huber, 2010) temperature increases would translate to more radical changes. For example, with approximately 7.2°C of warming, annual minimum pressures would be elevated to the *current* annual mean, effectively removing the additional low-oxygen challenge inherent with wintertime climbs. A climate 7.2°C warmer than pre-industrial would also raise the annual mean air pressure to 346 hPa—a value encountered around 300 m below the summit in the current climate on a typical May day. On the other hand, annual minimum pressures could be expected to drop below the ∼302 hPa threshold for an oxygenless summit (see Methods) in a climate 4.9°C colder than present. Although such cooling is implausible for many millennia to come (Collins et al., 2013; Herrero et al., 2014), temperatures were approximately 5–6°C cooler than present during the Last Glacial Maximum (Snyder, 2016). Our results therefore suggest that a hypothetical oxygenless climb to 8,850 m on Mt. Everest may have been occasionally beyond the limits of human aerobic capacity as recently as 19–26.5 thousand years ago (Clark et al., 2009).

The temperature sensitivities of summit pressure diagnosed by CMIP5 are supported by the general consistency with trends in the 1979–2019 reconstruction. Moore and Semple (2009) noted a similar rate of change in summit air pressure of 0.2–0.3 hPa decade^−1^ using the NCEP reanalysis (1948–2008; Kistler et al., 2001). However, the authors then suggested that the implied 2–3 hPa increase from 1958 to 2048 would increase VO_2_ max by ∼10%, far greater than the sensitivity we report here. This discrepancy seems to be because Moore and Semple (2009) did not adjust for the fact that the partial pressure of oxygen changes by ∼0.21 hPa for every 1 hPa increase in (total) air pressure (see Oxygen Availability and VO_2_ max in Methods), meaning they overestimate the impact on VO_2_ max by approximately a factor of five.

The large amount of warming required to elevate annual minimum pressures to the annual mean reflects the substantial seasonal cycle in summit air pressure for the current climate, with pressure in the depths of winter on average 16 hPa below values during the height of the monsoon. This range is consistent with previous estimates of seasonality and highlights how variable the aerobic challenge of an oxygenless ascent can be across a typical year (West et al., 1983b). We noted, however, that climbers succeeding with such attempts have been exposed only to a narrow sample of the conditions that are possible. This is mostly because oxygenless summits have been restricted to the relatively high-pressure months of May and October, but also because mountaineers exhibit a preference for climbing during episodes of relatively high pressure for the time of year, as reported elsewhere (Firth et al., 2008). The result is that, although across oxygenless summits the minimum VO_2_ max was reduced by 8.1% relative to its peak, the minimum pressure event in February 1993 (309 hPa) would result in a VO_2_ max 15.9% lower still, slowing the potential climbing rate by almost another third. Even though this February low-pressure extreme was above the theoretical ∼302 hPa threshold, the potential for such considerable increases in aerobic difficulty questions whether oxygenless summits are *always* within the grasp of human physiology under the current climate. The challenge of climbing Mt. Everest without supplemental oxygen in winter has been raised before but based on ∼324 hPa to characterize summit pressure (Garrido et al., 2019; West, 1999). Our reconstruction emphasizes that this value may be an appropriate descriptor of average conditions, but highlights the possibility of much lower pressures still, agreeing with previous research in noting the importance of variability (weather) around the mean (Moore and Semple, 2004, 2011; Moore et al., 2012).

The possibility of such challenging aerobic conditions has immediate practical consequences due to the renewed interest in wintertime oxygenless summits (Benavides, 2019, 2020). There are two observations from our analysis that may improve climber safety here. The first is the speed at which extreme low-pressure events can arrive. In agreement with Moore and Semple (2004) we noted declines in the order of ∼10 hPa in under four days, attributed to traveling upper-level waves embedded in the subtropical jet stream. This fall is equivalent to the difference in mean air pressures between May and January, occurring in the time it takes to climb from base camp to the summit and underscoring the importance of weather forecasts for climber safety. However, our second observation highlights that extreme low-pressure events are *not* associated with strong wintertime winds, which is the parameter usually employed to identify suitable weather windows for climbing (Peplow, 2004). As expected from the physical drivers of air pressure variation at high altitude, we do observe a strong correlation between reconstructed pressure and estimated temperature, but extreme cold is generally considered less of a barrier to wintertime mountaineering on Mt. Everest (Ogata, 1995). Our findings therefore strongly suggest that those attempting to make oxygenless ascents in winter should explicitly consult forecasts of summit air pressure. Given that wintertime pressure can occasionally even exceed levels more typical of the most popular climbing month of May, an oxygenless ascent of Mt. Everest in the depths of winter need not even push the physiological frontier if the weather window can be chosen wisely.

Dedicated forecasts of oxygen availability (through air pressure) could also pay dividends for mountaineers attempting to climb without supplemental oxygen in more popular climbing months. Although our results indicated that variability is suppressed relative to the winter, changes in May air pressure, for example, can still drive physiologically relevant reductions in VO_2_ max (Moore and Semple, 2004, 2006), important for the safety and success of high-elevation mountaineers (Richalet et al., 1988). Future research should explore this potential, as initial assessments indicate that numerical weather forecasts can skillfully capture atmospheric variability on Mt. Everest's upper slopes (Matthews et al., 2020). Added impetus for this development may be provided if future research determines that oxygen availability has a detectable influence on mountaineering success and safety, perhaps including for those climbing *with* supplemental oxygen (Fontanarosa et al., 2000; Huey et al., 2020).

To conclude, we have provided the most comprehensive assessment yet of oxygen availability at the summit of Mt. Everest, including quantifying its sensitivity to climate change. The results highlight that plausible amounts of warming from greenhouse gas emissions could potentially lead to physiologically relevant increases in oxygen availability, albeit to a lesser extent than indicated by previous research. This interesting consequence may be a powerful means to engage the wider public in climate change, but it is less practically relevant than the substantial seasonal and weather-induced variability in summit pressure confirmed by our analysis. The potential for rapid transitions to low pressures, close to human physiological limits, underscores the need for careful deployment of weather forecasts to help ensure the safety of those pushing the envelope on Mt. Everest.

### Limitations of the Study

There are several caveats that should be acknowledged when interpreting our study. First, it is recognized that reanalyses products are subject to temporal inhomogeneities, as the number and type of observations assimilated changes with time (Schneider and Fogt, 2018; Thorne and Vose, 2010). The reanalysis product used here to reconstruct summit air pressures (ERA5) starts in 1979, so it is not prone to any shocks from the widespread introduction of upper-air observations in the 1940s and 1950s (Stickler et al., 2009) or from the beginnings of satellite data assimilation in the late 1970s (Kistler et al., 2001). However, earlier years in ERA5 do suffer from far fewer observations of all forms (Hersbach et al., 2020), meaning that the uncertainties presented for our air pressure reconstruction are likely conservative in the earlier part of the record.

Second, the 21 CMIP5 models we used in our analysis represent, on average, warming up to 4.5°C above the 1981–2010 baseline. Projections of summit pressure for climates warmer still (e.g. 7.2°C above pre-industrial, as discussed in the text) or much cooler than 1981–2010 (e.g. approaching the temperatures of the Last Glacial Maximum) therefore require considerable extrapolation beyond the range of climate change scenarios assessed here. The projections of summit air pressure under such very different climates results should therefore be treated with caution.

We also highlight that, although we used 302 hPa as the lower limit for an oxygenless ascent, variability in individuals' aerobic fitness means that this threshold is not static. The 302 hPa value we applied is representative of very fit mountaineers, with a sea level VO_2_ max of approximately 57 mL kg^−1^ min^−1^ (Bailey, 2001; Pugh et al., 1964; Sutton et al., 1988; West et al., 1983a); individuals with higher sea level VO_2_ max (including some climbing Sherpa: Brutsaert, 2008; Garrido et al., 1997; Gilbert-Kawai et al., 2014) may be capable of oxygenless ascents at air pressures below 302 hPa. Our estimates of climbing rates mentioned in the text are similarly representative of this sea-level VO_2_ max and may underestimate the speeds that some climbers are capable of.

### Resource Availability

#### Lead Contact

Tom Matthews, Department of Geography and Environment, Loughborough University, UK, t.matthews@lboro.ac.uk.

#### Materials Availability

The study generated no new materials.

#### Data and Code Availability

The observational data from the automatic weather stations on Mt. Everest are available from National Geographic: https://www.nationalgeographic.org/projects/perpetual-planet/everest/weather-data/

Hourly ERA5 reanalysis data on pressure levels can be obtained freely from the Copernicus Climate Data Store, available here: https://cds.climate.copernicus.eu/cdsapp#!/dataset/reanalysis-era5-pressure-levels?tab=form.

The CMIP5 data (see Supplementary Information Table S1 for the models used) can be accessed freely through the Earth System Grid Federation (https://pcmdi.llnl.gov/mips/cmip5/data-access-getting-started.html).

The reconstructed summit air pressures for all oxygen ascents are available here: https://docs.google.com/spreadsheets/d/14IAtgzZt36YudrhHN0tAPWS4-ddyWf_9IfZx-Yy8kQQ/edit?usp=sharing.

Code used in the analysis is accessible on GitHub: https://github.com/climatom/OneEarth_Everest_O2.

## Methods

All methods can be found in the accompanying Transparent Methods supplemental file.

## Supplemental Information

Document S1. Transparent Methods, Figure S1, and Table S1
