# Supplementary material for: Into Thick(er) Air? Oxygen Availability at Humans' Physiological Frontier on Mount Everest
Source: iScience. 2020 Nov 20;23(12):101718. doi: 10.1016/j.isci.2020.101718 (PMC7756134; doi:10.1016/j.isci.2020.101718)
Supplement: Document S1. Transparent Methods, Figure S1, and Table S1 [file mmc1.pdf]

iScience, Volume *23*

Supplemental Information

Into Thick(er) Air? Oxygen Availability at Humans' Physiological Frontier on Mount Everest

Tom Matthews, L. Baker Perry, Timothy P. Lane, Aurora C. Elmore, Arbindra Khadka, Deepak Aryal, Dibas Shrestha, Subash Tuladhar, Saraju K. Baidya, Ananta Gajurel, Mariusz Potocki, and Paul A. Mayewski

Supplemental Information

Supplemental Figures and Tables

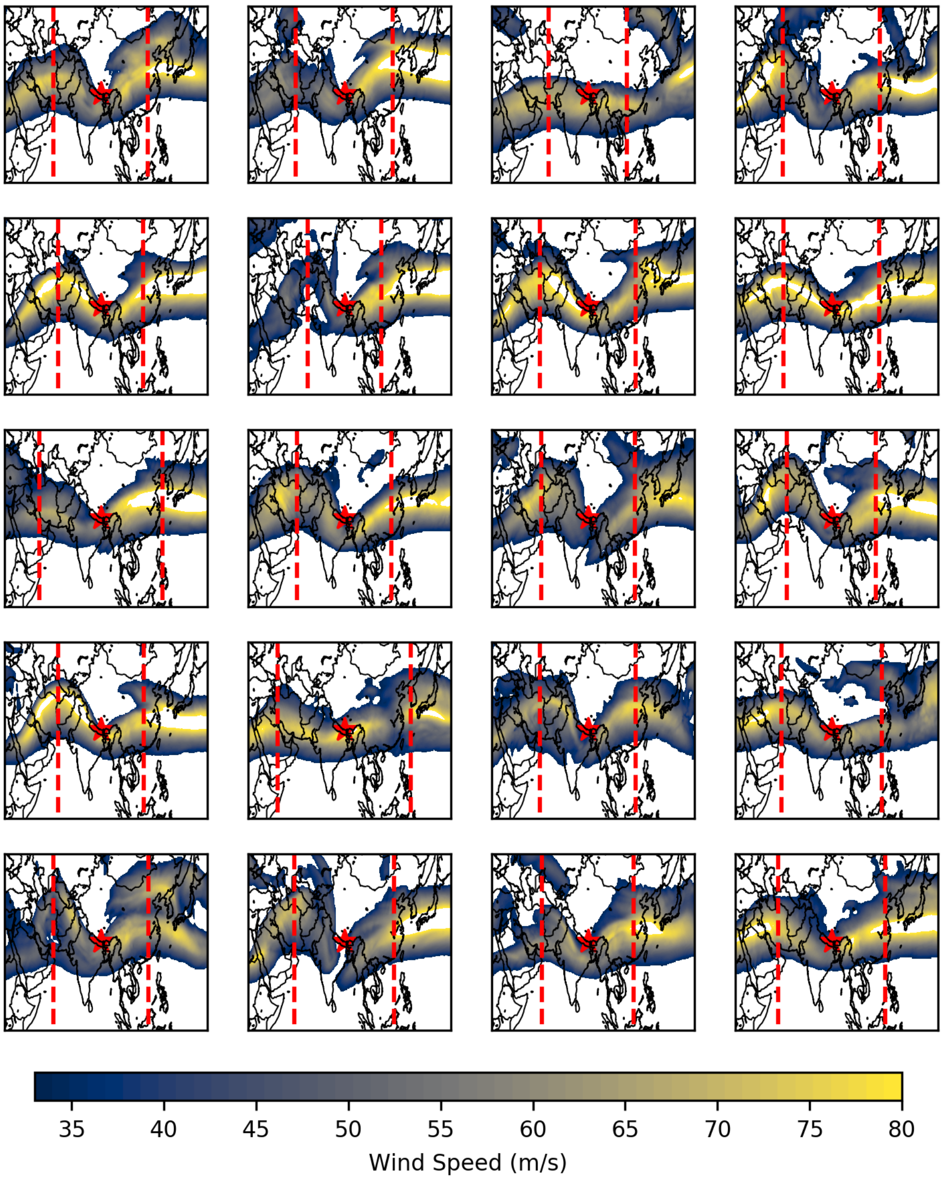

Figure. S1: Atmospheric circulation during the 20 events of lowest air pressure on the summit of Mount Everest, related to Fig. 3A. Colour ramp shows the wind at the 250 hPa pressure level, and the dotted red lines indicate the position of the wave crests identified by the algorithm described in the Transparent Methods (Atmospheric Circulation During Low Pressure Events).

Institute	Model
IPSL	IPSL-CM5B-LR
CMCC	CMCC-CESM
MIROC	MIROC-ESM-CHEM
LASG-CESS	FGOALS-g2
CCCma	CanESM2
BNU	BNU-ESM
NCC	NorESM1-M
BCC	bcc-csm1-1-m
NOAA-GFDL	GFDL-CM3
NOAA-GFDL	GFDL-ESM2G
MRI	MRI-CGCM3
MIROC	MIROC5
MOHC	HadGEM2-CC
IPSL	IPSL-CM5A-LR
MPI-M	MPI-ESM-MR
CMCC	CMCC-CMS
MIROC	MIROC-ESM
CMCC	CMCC-CM
CSIRO-BOM	ACCESS1-0
CSIRO-BOM	ACCESS1-3
IPSL	IPSL-CM5A-MR

Table S1. CMIP5 models used in the analysis, related to Fig 5 (C and D). Note that for each model we employed ensemble member R1i1p1 of the RCP8.5 experiment.

Transparent Methods

Estimation of Mt. Everest Summit Air Pressure

We use observations from the South Col (7,945 m) and Balcony (8,430 m) automatic weather stations (AWSs) deployed on the main southern (Nepalese) climbing route during the 2019 National Geographic and Rolex Perpetual Planet Everest Expedition (Matthews et al., 2020). For the South Col, hourly mean air pressure data were employed from 06:00 UTC May 22, 2019 to 06:00 UTC July 1, 2020. At the

Balcony, the record used is shorter (01:00 UTC May 23, 2019 to 05:00 UTC January 20, 2020), as that station stopped transmitting during the 2019/2020 winter.

To reconstruct air pressure over the longer-term, we used the ERA5 reanalysis from the European Centre for Medium Range Weather Forecasting (Hersbach et al., 2020). We extracted hourly geopotential height, air temperature and wind speed on pressure surfaces for the full period of data availability at the time of analysis (00:00 UTC on January 1, 1979 to 21:00 UTC on June 20, 2020) and then bi-linerarly interpolated these data to the location of Mt. Everest's summit (27.98 °N, 86.93 °E). Air pressure was also interpolated to the location of the longer-running South Col AWS (7,945 m), where an empirical quantile mapping procedure was used to remove systematic bias (Gudmundsson et al., 2012):

$$P_c = f(P_r, x, y) \quad \text{Eq. 1}$$

Where f is a function that interpolates to find the corrected value of the South Col reanalysis air pressure (P_c), given the uncorrected reanalysis data (P_r) and ordered samples *x, y*:

$$x = g^{-1}(P_{r-cal}, q) \quad \text{Eq. 2}$$

and:

$$y = g^{-1}(O, q) \quad \text{Eq. 3}$$

Where $P_{r-cal}$is the air pressure subset of reanalysis data that overlaps with the AWS observations (06:00 UTC May 22, 2019 to 21:00 UTC on June 20, 2020); O is the observed air pressure at the South Col AWS; $q$is vector of quantiles (0.01 to 0.99 in increments of 0.01); and g is the cumulative distribution function. Note that the interpolation was only applied to values of R_r within the range of P_{r-cal}; values outside were adjusted with:$P_c = P_r + \{g^{-1}(O, k) - g^{-1}(P_{r-cal}, k)\}$, where k adopts values of 0.01 and 0.99 when $R_r$is below and above the range of P_{r-cal}, respectively.

Air pressures were then estimated at the summit of Mt. Everest according to the hypsometric equation (Stull, 2015):

$$P_2 = P_1 \times exp(\frac{z_1 - z_2}{a\,\overline{T_v}})$$

Eq. 4

where P_x denotes air pressure at height z_x (m), a is constant (29.3 m K^{-1}) and $\overline{T_v}$ is the mean virtual air temperature (K) between heights z_1 and z_2.

We rewrite Eq. 4 to get the gradient in (log) air pressure as a function of elevation ($\frac{ln(p_2)-ln(p_1)}{z_1-z_2} = \Delta ln(P)/\Delta z$):

$$\Delta ln(P)/\Delta z = a\overline{T_v}^{-1}$$

Eq. 5

Enabling air pressure at the summit (P_s) to be evaluated from (corrected) air pressure at the South Col:

$$P_s = P_c \times\ exp(\frac{903}{a\,\overline{T_v}})$$

Eq. 6

Where 903 (m) is the vertical separation between the South Col and the 8,850 m summit.

To enable application of Equation 6, it is necessary to know $\overline{T_v}$ between the South Col and the summit, which we estimated from:

$$\overline{T_v} = 0.5 \times (2 \times T_{col}\ +\ \Gamma \Delta z)$$

Eq. 7

where T_{col} is the ERA5 air temperature interpolated from pressure levels to the location of the South Col AWS; and Γ is the temperature lapse rate ($\Delta T/\Delta z$), obtained from the air temperature and geopotential height on the 300 and 400 hPa pressure surfaces (a conservative selection intended to bound the maximum pressure at the South Col and minimum pressure at the summit). Any biases in the renalysis $\overline{T_v}$ will, however, affect our assessment of the vertical (log) pressure gradient (Eq. 7). To correct for this, we used air pressures at the Balcony and South Col AWSs to estimate the hourly vertical gradient in log pressure, and regressed this on concurrent $a\overline{T_v}^{-1}$ for the overlapping period. Substitution

into Eq. 6 enables the summit pressure to be estimated using these empirically determined slope (β) and intercept (α) regression terms:

$$P_s = P_c\, exp(\frac{903}{\alpha + \beta(a\,\overline{T_v})})$$

Eq. 8

P_s was reconstructed for the complete calendar years 1979-2019. Day of year quantities presented in the text and Fig. 2 were computed by first computing the statistic for the respective day (1-366), and then smoothing these values via convolution with a Gaussian filter set to have a standard deviation of seven days.

Oxygen Availability and VO_2 max

Air pressure was converted to VO_2 max by first calculating the partial pressure of inspired oxygen (P_{io}):

$$P_{io} = 0.2095 \times (P_s - 62.9)$$

Eq. 9

where 62.9 (hPa) is the saturation vapour pressure at the human body's core temperature of 37 °C, and 0.2095 represents the volume fraction of oxygen in the atmosphere (Wallace and Hobbs, 1977).

We then rearranged the regression equation of Bailey (2001) (who synthesised the results of Pugh et al., 1964; Sutton et al., 1988; and West et al., 1983b) to obtain the aerobic capacity (VO_2 max, ml kg^{-1} min^{-1}) of acclimatized individuals as a function of P_{io}(in hPa):

$$VO_2max = \frac{ln(P_{io} \times 0.750) - 3.25}{0.0308}$$

Eq. 10

Equations 9 and 10 therefore enable changes in summit air pressure to be communicated in terms of aerobic impact -- the reduction in VO_2 max due to declining oxygen availability. Bailey (2001) estimate a minimum of 12.25 ml kg^{-1} min^{-1} (3.5 metabolic equivalent expenditures: METs) is required to safely ascend Mt. Everest, assuming summertime conditions, and that climbers are operating at around 85 % of their VO_2 max. Inserting this value into Eq. 10 and solving for P_s (via Eq. 9) yields a threshold air pressure of 302 hPa at the summit for Mt. Everest to be climbable without supplemental oxygen. As discussed in the Limitations (main text), we note that variation in VO_2 max amongst mountaineers is not

accounted for here, and the 302 hPa threshold we identify is representative of fit mountaineers. Some climbers (including elite climbing Sherpa; Brutsaert, 2008; Garrido et al., 1997; Gilbert-Kawai et al., 2014) will have even higher VO_2 max than determined by Eq. 10, and may therefore be able to complete an oxygenless summit at air pressures below 302 hPa.

Atmospheric Circulation During Low Pressure Events

Low pressure events were defined as the 20 lowest hourly air pressure values, separated from other minima by at least two days. To explore atmospheric circulation during these events, we composited the height of the 300 hPa surface (the pressure level closest to the summit of Mt. Everest), and wind velocity at the 250 hPa surface (where the subtropical jet stream is normally located; Ren et al., 2011). Inspection of the composite (Figure 3B), and of the circulation during the individual events (Supplementary Information, Figure S1), indicated the presence of a well-defined upper-level trough with its axis centred at the longitude of Mt. Everest. For each of the 20 waves we calculated the zonal distance from Mt. Everest's summit to the well-defined ridge crest often found to the east, whose location was identified as the longitude with maximum geopotential height along 28 °N, 30-86.9 °E. Doubling this zonal distance provided an estimate of the wavelength (λ) for each of the waves. The time taken for these waves to transit Mt. Everest was estimated using their phase speed (c), calculated assuming barotropic instability, which is a reasonable approximation away from the polar front (Stull, 2015):

$$c = -\frac{2\Omega}{R} cos(\theta) \times \left(\frac{\lambda}{2\pi}\right)^2 + U_{500}$$

Eq.11

where Ω is the Earth's angular velocity (7.29 $\times 10^{-5}$ radians s^{-1}), R is the Earth's radius (6.371 $\times 10^{6}$ m), θ is the latitude (set to 28 °N here), and U_{500} is the mean wind velocity (m s^{-1}) at the 500 hPa level, averaged over the rectangular region 20-40 °N, 30-150 °E. The time taken for the wave trough to arrive/depart Mt. Everest was then evaluated as $\frac{\lambda}{2c}$. It is this time horizon which is marked with vertical red lines in Figure 3A.

Air Pressure and Oxygen Availability During Summit Climbs

The Himalayan Database (Hawley and Salisbury, 2007) provides a comprehensive history of Mt. Everest mountaineering. We used it here to identify successful climbs without supplemental oxygen over the period 1979-2019, extracting reconstructed summit air pressure for the hour that each climber reached the peak. For 19 of these 208 ascents, the exact time was not recorded, so we estimated the summit pressure at the time of ascent using a Gaussian weighted average (with a standard deviation of 3.5 h) centred at 12:00 Nepal Time (NPT) on the day of the successful climb. These choices reflect the mean and standard deviation of summit times across the 189 records that recorded this information.

Estimates of Work Rate and Climbing Speed

Estimates of maximum work rate (W) were informed by the empirical relationship outlined by West et al. (1983b). We digitized the regression line plotted in their Fig. 2, extracting the slope (β) and intercept (α) coefficients to enable conversion between quantities.:

$$W = \beta \times VO_2 max + \alpha \qquad \text{Eq. 12}$$

The value of β and α were, respectively, determined to be 41.54 kg^2 m ml^{-1}, and -255.96 kg m min^{-1}. Before applying Eq. 12, we reduced each VO_2 max by 15 % to acknowledge that mountaineers likely climb at 85 % of their VO_2 max (Bailey, 2001). W is in units of kg m min^{-1}, and the speed of vertical ascent (m min^{-1}) can be isolated if the mass (kg) of the mountaineer is prescribed. Following West et al. (1983b), we set the mass of the hypothetical climber (including equipment) to 100 kg. Note that because W is a function of VO_2, work rates and climbing speeds should be interpreted as representative of fit mountaineers, but not necessarily elite climbing Sherpa (see Limitations in main text).

The Impact of Climate Change on Summit Pressure

To summarise changes in summit pressure over the period of ERA5 reconstruction (1979-2019), we computed the monthly minimum, mean, and maximum summit pressures. Rates of change were then summarised for these quantities using the Theil-Sen slope estimation method (Sen, 1960; Theil, 1950). The respective trends were termed *significant* if zero lay outside the 95 % confidence interval of the slope estimate.

We used daily mean pressure level CMIP5 output from 21 models forced by the RCP8.5 experiment (Taylor et al., 2011) to determine the sensitivity of Mt. Everest summit air pressure to global mean warming. For each model (listed in Table S1) the same interpolation method applied to the ERA5 reanalysis data to estimate summit pressure was employed. We also extracted the respective near-surface global mean air temperature (T_g) simulated by the corresponding model.

The sensitivity of Mt. Everest summit pressure to changes in $T_g$was then evaluated using the change factor approach (Osborn et al., 2016). Briefly, this comprised (i) estimating the modelled sensitivity of summit pressures to changes in global mean temperature; (ii) multiplying this sensitivity by a prescribed temperature perturbation; and (iii) adding this result to air pressures in the baseline climate. We achieved (i) by first smoothing CMIP5 P_s and T_g with a running 30-year mean filter, and then regressing P_s upon T_g. Regressions were performed on a seasonal basis, assessing the sensitivity of the (30-year mean) monthly minimum, maximum, and mean summit pressures to climate warming. The results from this analysis were a 21-member ensemble of regression slope coefficients indicating the sensitivity (β^{stat}_{month}, hPa °C^{-1}) to statistic ^{stat} (minimum, maximum or mean) in the respective $_{month}$. The monthly stratification of the regression coefficients was warranted because for mean and minimum summit pressure, a single factor ANOVA indicated significant differences across months (p < 0.01). Evidence for different sensitivities of maximum summit pressure across months was weaker (p = 0.13), but we kept the monthly stratification to be consistent across statistics.

For each model, steps (ii) and (iii) were achieved by transforming the sensitivities (β^{stat}_{month}) to absolute values of air pressure ($\widehat{P^{stat}_{s_{month}}}$) given prescribed changes (Δ) to $T_g$through:

$$\widehat{P^{stat}_{s_{month}}} = \beta^{stat}_{month}\Delta T_g + P^{stat}_{s_{month}}$$

Eq. 13

where $P^{stat}_{s_{month}}$is the 1981-2010 statistic for the respective month in the ERA5 reconstructed summit air pressure series. The median, 5th, and 95th percentiles of β^{stat}_{month}across the model simulations were used to indicate, respectively, the central estimate and uncertainty in application of Eq. 13. Annual means, minima and maxima were then evaluated for the respective climates by calculating the relevant statistic from these transformed series. We characterized departures from the 1981-2010 global mean air

temperature using the HadCRUT4 dataset (Morice et al., 2012). Note that according to these data, this period was 0.60 °C warmer than preindustrial, defined here as 1850-1879.

Supplemental References

Gudmundsson, L., Bremnes, J.B., Haugen, J.E., Engen-Skaugen, T., 2012. Technical Note: Downscaling RCM precipitation to the station scale using statistical transformations – a comparison of methods. Hydrol. Earth Syst. Sci. 16, 3383–3390. https://doi.org/10.5194/hess-16-3383-2012

Hawley, E., and R. Salisbury. "The Himalayan Database: The Expedition Archives of Elizabeth Hawley," 2007. https://www.himalayandatabase.com/.

Osborn, T.J., Wallace, C.J., Harris, I.C., Melvin, T.M., 2016. Pattern scaling using ClimGen: monthly-resolution future climate scenarios including changes in the variability of precipitation. Clim. Change 134, 353–369. https://doi.org/10.1007/s10584-015-1509-9

Ren, X., Yang, X., Zhou, T., Fang, J., 2011. Diagnostic comparison of wintertime East Asian subtropical jet and polar-front jet: Large-scale characteristics and transient eddy activities. Acta Meteorol Sin 25, 21–33. https://doi.org/10.1007/s13351-011-0002-2

Sen, P.K., 1960. On Some Convergence Properties of U-Statistics. Calcutta Statistical Association Bulletin 10, 1–18. https://doi.org/10.1177/0008068319600101

Theil, H., 1950. A Rank-Invariant Method of Linear and Polynomial Regression Analysis. Koninklijke Nederlandse Akademie var Wetenschappen Proceedings 53, 386–392. (Part 1), 521–525 (Part 2), 1397–1412 (Part 3).

Wallace, J.M., Hobbs, P.V., 1977. Atmopsheric Science: An Introductory Survey. Academic Press, New York.

West, J.B., Lahiri, S., Maret, K.H., Peters, R.M., Pizzo, C.J., 1983b. Barometric pressures at extreme altitudes on Mt. Everest: physiological significance. J Appl. Physiol. Respir. Environ. Exerc. Physiol. 54, 1188–1194. https://doi.org/10.1152/jappl.1983.54.5.1188
